# Fat Mass and Obesity-Associated (FTO) Gene Polymorphisms Are Associated with Physical Activity, Food Intake, Eating Behaviors, Psychological Health, and Modeled Change in Body Mass Index in Overweight/Obese Caucasian Adults

**DOI:** 10.3390/nu6083130

**Published:** 2014-08-06

**Authors:** Janetta Harbron, Lize van der Merwe, Monique G. Zaahl, Maritha J. Kotze, Marjanne Senekal

**Affiliations:** 1 Division of Human Nutrition, Department of Human Biology, Faculty of Health Sciences, University of Cape Town, Private Bag X3, Observatory 7925, South Africa; E-Mail: marjanne.senekal@uct.ac.za; 2 Division of Molecular Biology and Human Genetics, Faculty of Medicine and Health Sciences, Stellenbosch University, Tygerberg 7505, South Africa; E-Mail: Lize@LizeStats.co.za; 3 Department of Statistics, University of the Western Cape, Private Bag X17, Bellville 7535, South Africa; 4 Department of Genetics, Faculty of Sciences, Stellenbosch University, Private Bag X1, Matieland 7602, South Africa; E-Mail: mjulies@sun.ac.za; 5 Division of Anatomical Pathology, Department of Pathology, Faculty of Medicine and Health Sciences, Stellenbosch University, P.O. Box 19063, Tygerberg 7505, South Africa; E-Mail: maritha@sun.ac.za

**Keywords:** fat mass and obesity associated gene, *FTO* gene, eating behavior, depression, psychological well-being, dietary intake, physical activity, BMI, obesity

## Abstract

The fat mass and obesity-associated (*FTO*) gene is currently recognized as the most robust predictor of polygenic obesity. We investigated associations between the *FTO* rs1421085 and rs17817449 polymorphisms and the *FTO* rs1421085–rs17817449 haplotype and dietary intake, eating behavior, physical activity, and psychological health, as well as the effect of these associations on BMI. *N* = 133 treatment seeking overweight/obese Caucasian adults participated in this study. Genotyping was performed from whole blood samples. Weight and height was measured and a non-quantified food frequency questionnaire was completed to assess food group intake. Validated questionnaires were completed to assess physical activity (Baecke questionnaire), psychological health (General Health questionnaire, Rosenburg self-esteem scale and Beck Depression Inventory), and eating behavior (Three Factor Eating questionnaire). The risk alleles of the *FTO* polymorphisms were associated with poorer eating behaviors (higher hunger, internal locus for hunger, and emotional disinhibition scores), a higher intake of high fat foods and refined starches and more depressive symptoms. The modeled results indicate that interactions between the *FTO* polymorphisms or haplotypes and eating behavior, psychological health, and physical activity levels may be associated with BMI. The clinical significance of these results for implementation as part of weight management interventions needs further investigation.

## 1. Introduction

Obesity has reached epidemic proportions and is still escalating at an alarming rate world-wide, affecting children and adults in both developed and developing countries [1,2]. The etiology of obesity is multi-factorial and any combination of environmental and lifestyle factors may interact with multiple genetic polymorphisms to result in the condition [3,4]. To date the fat mass and obesity-associated (*FTO*) gene stands out as the most robust and significant genetic contributor to polygenic obesity [5,6]. The risk alleles of several *FTO* polymorphisms located within a 47 kb linkage disequilibrium (LD) block encompassing sections of intron 1 and exon 2 of *FTO* have been associated with obesity and a higher BMI [7,8,9]. In genome-wide association studies the strongest associations with weight related phenotypes were reported for the *FTO* rs9939609 [8], rs9930506 [9], rs1421085, rs17817449, and rs1121980 [7] polymorphisms. However, the cluster of afore-mentioned polymorphisms is intronic and their physiological effects contributing to obesity development remain to be identified [10].

*FTO* encodes a Fe(II)- and 2-oxoglutarate (OG) dependent nucleic acid demethylase that localizes to the nucleus of cells [11,12]. However, the exact structure of the FTO protein [13] as well as its physiological function and role in obesity development still need to be elucidated [14]. The results of *in vitro* experiments point to the possibility that *FTO* exerts gene regulation at RNA level in humans, as it catalyzes the demethylation of 3-methylthymine in single-stranded DNA, 3-methyluracil in RNA [11,13,15] and N6-methyladenosine in nuclear RNA [16].

*FTO* is expressed in the cell nucleus of almost all human tissues [7]. The highest expression levels are found in the brain, specifically in the arcuate nucleus of the hypothalamus, which is known to play a major role in controlling energy homeostasis and eating behavior [8]. A possible role in energy homeostasis is supported by studies performed on humans, mice and rodents that have shown that *Fto* mRNA expression is regulated by food intake [11,17,18,19], circulating glucose levels [20], weight status [21,22] and energy expenditure [18,23]. In human and mouse cell lines, FTO expression is reversibly influenced by essential amino acid deprivation and replacement [24]. Furthermore, Pitman *et al.* [25] illustrated that knockdown of *FTO* expression increased ATP concentrations in neuronal cells, but decreased ATP concentrations in adipocytes, implying a mechanism of cell-specific control over energy production.

Research in mouse models shows that knockout of *Fto* results in normal embryonic development, but high postnatal lethality, growth retardation and lower weight and adiposity measures [23,26,27]. Similarly, adult onset *Fto* knockout mice also experience reduced weight and lean mass initially. However, thereafter their weight tended to converge with that of wild-type mice due to increases in body fat mass [27]. Fischer *et al.* [23] indicated that *Fto* knockout mice were protected from diet-induced obesity throughout their entire life span when consuming a high fat diet, while a high fat intake augmented weight gain in the mice with FTO overexpression [26]. Overexpression of FTO also seemed to be associated with higher food intakes and consequent weight and fat mass gains when expenditure and physical activity levels remained unchanged [26]. A number of possible but inconclusive mechanisms for FTO’s role in explaining these findings have been proposed. These include that FTO may influence protein and/or fat utilization [27], as well as energy expenditure and adrenalin levels [23,28].

Results from work in humans are also not yet conclusive, with some reporting a link between the risk alleles and increased energy expenditure [29,30], whereas others failed to show any association [31,32,33,34]. The risk alleles have also been associated with poorer eating behaviors [7,8,9], higher food, energy and fat intakes and specific preference for energy-dense foods with high fat content [29,32,34,35,36,37]. Although these studies mostly investigated the rs9939609 polymorphism, McCaffery *et al.* [37] suggest that the rs1421085 polymorphism may better illustrate the effect of the *FTO* locus on dietary intake and patterns, as the associations they found between the latter variables and the risk alleles of rs1421085 were more consistent compared to the other *FTO* polymorphisms investigated (rs9939609, rs3751812, rs9922708). However, it needs to be noted that Stutzman *et al.* [38] found no association between the rs1421085 polymorphism and eating behavior or meal size and snacking patterns in Caucasians [38]. There is a paucity of information on the association between *FTO* rs17817449 and lifestyle related variables. Further research is clearly necessary to elucidate the associations of *FTO* polymorphisms with eating behavior, dietary intake, and energy expenditure.

The objectives of this study were firstly to investigate the association between the *FTO* rs1421085 and rs17817449 polymorphisms as well as the *FTO* rs1421085-rs17817449 haplotype and dietary intake, eating behavior, physical activity, and psychological health in treatment seeking overweight/obese subjects, and secondly to determine the effect of the interaction between these *FTO* polymorphisms as well as haplotype and the mentioned variables on BMI. Please note that we refer to the C-allele of the *FTO* rs1421085 polymorphism and the G-allele of the *FTO* rs17817449 polymorphism as the risk alleles, and the T-allele of both polymorphisms as the non-risk alleles as previously described in the literature [7,30].

## 2. Experimental Section

### 2.1. Study Participants

The study population consisted of treatment seeking overweight and obese Caucasian subjects who were South African citizens and were residing in Cape Town and surrounding areas. In this study “Caucasian” refers to individuals of European descent, mainly from Dutch, French, German, and British origin. Subjects were recruited by means of advertisements placed in local newspapers, the e-mail bulletins of local universities, and by word of mouth to participate in a weight loss intervention to investigate the association between weight loss and genotype. All subjects attended an individual recruitment interview with a registered dietitian to provide detailed information regarding the project, take anthropometric measurements, and complete a number of questionnaires, including questions on the inclusion and exclusion criteria of the study. To be included, subjects had to be between the ages of 25 and 40 years old with a BMI ≥ 27 kg/m^2^. Those who were pregnant or breastfeeding, had a history of eating disorders, serious psychiatric illnesses or drug or alcohol abuse were excluded. Appointments for two follow-up sessions were made during which the rest of the questionnaires were completed and a blood sample was taken. A cross-sectional sample of *n* = 133 overweight/obese subjects was assessed. The study was approved by the Health Research Ethics Committee of Stellenbosch University and signed informed consent was obtained from all subjects.

### 2.2. Genotyping

Blood was drawn by trained phlebotomists in BD vacutainers with EDTA. A modified protocol by Miller *et al.* [39] was used to extract DNA from the whole blood samples. The polymorphisms were genotyped using PCR amplification in a GeneAmp^®^ 2700 PCR System (Applied Biosystems, Foster city, CA, USA). The volume of each PCR reaction was 25 μL consisting of 50 ng DNA, 0.2 mM of each dNTP (dATP, dCTP, dGTP, dTTP), 10 pmoL of each primer, 2 mM magnesium chloride (MgCl_2_), *Taq* polymerase and 1× *Taq* buffer. The following primers, manufactured by Integrated DNA Technologies (Leuven, Belgium), were used: 5′-TAGTAGCAGTTCAGGTCCTAAGGCGTG-3′ (forward) and 5′-CAGATTAAGGTGATGGGTTG-3′ (reverse) for the *FTO* rs1421085 polymorphism (designed using *In Silico*); 5′-AGGACCTCCTATTTGGGACA-3′ (forward) and 5′-AGCTTCCATGGCTAGCATTA-3′ (reverse) for the *FTO* rs17817449 polymorphism [30]. The PCR cycles consisted of an initial denaturation at 95 °C for 5 min, followed by 35 cycles of denaturation at 95 °C for 30 s, annealing at 55 °C for 45 s and elongation at 72 °C for 30 s. The final extension step occurred at 72 °C for 10 min. A mixture of 5 μL of each PCR product and 5 μL cresol red loading buffer (2 mg/mL cresol red and 35% (w/v) sucrose) was loaded onto a 1% (w/v) horizontal agarose gel (2 g agarose in 200 mL 1× TBE (90 mM Tris-HCl, 90 mM boric acid (H_3_BO_3_) and 0.1 mM EDTA, pH 8.0) and 0.01% ethidium bromide (EtBr)). Electrophoresis was performed at 120 V for one hour in 1× TBE buffer solution and then visualized by ultraviolet light transillumination (GeneSnap MultiGenius Bio Imaging System, Syngene, Synoptics, Cambridge, UK). A 100 bp ladder was also loaded to confirm PCR amplification products of the correct fragment size, *i.e.*, 240 bp (*FTO* rs1421085) and 828 bp (*FTO* rs17817449).

Digestion of the PCR products was performed using restriction fragment length polymorphism (RFLP) analysis in 20 μL reactions containing 10 μL PCR product, 1× buffer and 1U *Mae*III for the *FTO* rs1421085 polymorphism or 2U *AlwN*I for the *FTO* rs17817449 polymorphism. The solution was incubated at 55 °C (*FTO* rs1421085) or 37 °C (*FTO* rs17817449) in a water bath overnight. The digested PCR products were loaded onto a 3% agarose gel at 120 V for 1 h (*FTO* rs1421085) or 1.5% Agarose gel at 120 V for 50 min and visualized by ultraviolet light transillumination (GeneSnap MultiGenius Bio Imaging System, Syngene). Digestion results for all three genotypes of both polymorphisms were confirmed by Inqaba biotec (Inqaba Biotechnical Industries, Pretoria, South Africa) using sequencing analysis, confirming analytical validity.

### 2.3. Anthropometry

Weight and height were measured by trained and standardized fieldworkers and body mass index (BMI) was computed as weight (kg)/height (m)^2^. Weight was measured in light clothing without shoes to the nearest 0.1 kg using a calibrated electronic scale with a 250 kg capacity (Physician scale, Scales 2000, Durban, South Africa). Height without shoes was measured to the nearest 0.1 cm with a stadiometer (Scales 2000, Durban, South Africa). The participants stood with their feet together and heels, buttocks, scapulae, and back of the head touching the vertical surface of the stadiometer [40].

### 2.4. Physical Activity

The validated [41,42,43] self-administered 16-item Baecke Questionnaire of Habitual Physical Activity [44] was used to measure physical activity at work (work index), sport during leisure time (sport index), and physical activity during leisure time, excluding sport (leisure-time index). Baecke *et al.* [44] reported good test-retest reliability for the work, sport, and leisure-time indices of 0.88, 0.81 and 0.74, respectively. The scores for the three indices were calculated as described by Baecke *et al.* [44]. Higher mean scores for each index reflect higher physical activity levels.

### 2.5. Dietary Intake

The aim of the dietary assessment was to investigate associations between the frequency of intake of four indicator food groups and genotype. These food groups were derived from the food list of a more comprehensive non-quantified food frequency questionnaire (FFQ) developed for this research. Although not validated, this FFQ was derived by a panel of experts from a generic FFQ developed by Steyn and Senekal [45] for South Africans, ensuring content and face validity. The four food groups included (1) high fat foods (21 items), (2) energy-dense snacks (8 items), (3) energy-dense drinks (4 items) and (4) refined bread and cereals (2 items). Frequency of intake of specific food items could be indicated as <1/month, 1–3 times/months, 1/week, 2–4/week, 5–6/week, 1/day, 2–3/day, 4–5/day, 6+/day. For data-analysis the mean daily frequency of intake of each of the four food groups was calculated.

### 2.6. Eating Behavior

Eating behavior was assessed with the validated [46,47] self-administered 51-item Three-Factor Eating Questionnaire (TFEQ) [48]. Scores for three dimensions (scales) of eating behavior, namely dietary restraint, disinhibition and perceived hunger [48] as well as different subscales (summarized in Table 1) [49,50] were calculated. Higher scores on scales and subscales reflect higher levels of restrained eating, disinhibited eating, and predisposition to hunger [51]. For our sample the Cronbach’s alpha was 0.78 for restraint, 0.81 for disinhibition, and 0.78 for perceived hunger, indicating good internal reliability.

**Table 1:** Summary, explanation, score range, and reported association with body mass index (BMI) of the scales and subscales of the three-factor eating questionnaire.

Eating Behavior Scale or Subscale	Explanation of Scale or Subscale	Score Range	Reported/ Expected Association with BMI	Reference
**Dietary restraint**	The extent to which food intake is cognitively restricted (by thought and will power) in order to control body shape and weight.	0 to 21	− or + *	[48,49,50]
Flexible control (consistent restraint)	A more gradual approach towards eating and dieting. Foods like sweets and treats or fattening foods are eaten but in smaller quantities, without feelings of guilt.	0 to 7	−	[49]
Rigid control (inconsistent restraint)	A dichotomous (all-or-nothing) approach towards eating and dieting. All sweets, treats, and fattening foods are avoided.	0 to 7	+	[49]
Strategic dieting behavior	Specific behaviors employed to control weight	0 to 4	− or + ^§^	[50]
Attitude to self-regulation (of eating)	Subjects’ general view on dietary intake and weight control.	0 to 5	+	[50]
Avoidance of fattening foods	Deliberate efforts to decrease fat content in the diet.	0 to 4		[50]
**Disinhibition**	The extent of inability to control food intake in response to the presence of (1) palatable food that may result in the over-consumption of food or (2) stimuli such as emotional stress or social eating cues that may result in the inability to resist food intake when not hungry	0 to 16	+	[48,49,50]
Habitual susceptibility to disinhibition	Circumstances predispose to recurrent disinhibition	0 to 5	+	[50]
Emotional susceptibility to disinhibition	Specific negative affective states such as emotional stress result in disinhibition	0 to 3	+	[50]
Situational susceptibility to disinhibition	Specific environmental cues result in disinhibition	0 to 5	+	[50]
**Perceived hunger**	The extent of food intake in response to susceptibility to general subjective feelings and perceptions of hunger and the behavioral consequences thereof	0 to 16	+	[48,52]
Internal locus for hunger	Type of hunger that is interpreted and regulated internally	0 to 6	+	[50]
External locus for hunger	Type of hunger that is regulated by external cues	0 to 6	+	[50]

(−) Negative association and (+) positive association. * Conflicting associations have been reported, also reflected in discussion of our results. ^§^ Negative association for those dissatisfied with their weight; positive association for those satisfied with their weight.

### 2.7. Psychological Health

The presence and severity of depressive symptoms was assessed with the validated [53,54] self-administered 21-item Beck Depression Inventory (BDI) [54]. Scores for each of the 21 items are summed to obtain a total score between 0 and 63. Higher scores reflect the presence of more severe depressive symptoms [54]. For our sample the Cronbach’s alpha for the BDI was 0.92, indicating excellent internal reliability.

General psychological well-being was assessed with the validated [55,56,57] self-administered 30-item General Health Questionnaire (GHQ) [56]. The questionnaire was developed to diagnose possible cases of non-psychotic psychiatric disorders and differentiates psychiatric patients as a general class from those who consider themselves to be well. A total score between 0 and 30 can be obtained. A higher score is indicative of poorer mental health and a greater inability to carry out one’s normal ‘healthy’ functions [55]. For our sample the Cronbach’s alpha for the GHQ was 0.87, indicating good internal reliability.

Self-esteem was assessed with the validated [58,59] 10-item self-administered Rosenberg Self-Esteem Scale (RSES) that was developed by Rosenberg [60] to measure global feelings of self-worth or self-acceptance. A total score between 10 and 40 can be obtained. Higher scores reflect a higher self-esteem [60]. For our sample the Cronbach’s alpha for the RSES was 0.87, indicating good internal reliability.

### 2.8. Statistical Analyses

For descriptive purposes, frequencies were calculated for categorical variables and means and standard deviations (Mean ± SD) for numerical variables, as they were found to be normally distributed (see Table 2). General linear models were used for all analyses, except for testing Hardy-Weinberg Equilibrium (HWE). Unadjusted tests were used for comparing baseline variables, and as significant gender differences were detected in a number of variables (results not shown), all further analyses were adjusted for gender.

Genotype distributions were tested for HWE using the exact test. Gender-adjusted linear models were used to test the association between lifestyle and psychological health variables and each *FTO* polymorphism using the (1) genotype model (comparing the three genotype groups), (2) dominant model (homozygous non-risk genotype *vs.* risk allele carriers), (3) recessive model (non-risk allele carriers *vs.* homozygous risk genotype) and (4) additive allelic model (counted as zero, one and two risk alleles). For these analyses only the significant differences of the best fitting model (smallest *p*-value) are reported in the results section (see Table 3). Effect sizes were calculated to describe each significant association.

Gender-adjusted linear models were also used to determine the effect of the interaction between each lifestyle and psychological health variable and (1) the genotype groups (categorized as non-risk, heterozygous, and risk genotypes) on BMI; and (2) the additive allelic variable (numerically coded as zero, one and two minor alleles) on BMI. In all cases where significant p-values for the effect of the interaction between **genotype** **groups** with a specific variable on BMI was found, a significant effect for the **additive** **allelic** **variable** interaction with the variable was also found. Therefore, to visualize significant results, a plot depicting each of the significant **additive** **allelic** **interactions** with a specified variable (*x*-axes) on BMI (*y*-axes) was drawn. On all plots, the symbols represent all (male and female) individual observed values and the regression lines show the expected relationships for a female for each genotype of the polymorphism under investigation. Due to gender adjustment of results, separate plots need to be constructed for each gender. As there were more females in the sample, we chose to depict regression lines for females to illustrate the results. The expected relationships between the lines would be exactly the same for males, their trio of graphs would just start at a different point on the y-axis. The plots reflect the modeled rate of change in BMI in response to a change in a specified variable for each genotype group. A significant additive allelic interaction indicates that the slopes (e.g., increase in BMI corresponding to a one-unit increase/decrease in the lifestyle or psychological health variable) of the regression lines of each genotype group differ significantly from each other. A positive interaction effect size indicates that the slope of the regression line for the heterozygotes is larger than for subjects with no risk alleles, and the slope for the subjects with two risk alleles is even larger. Thus, a negative interaction effect size indicates that the slope of the regression line for the heterozygotes is larger than of subjects with risk alleles, and the slope for the subjects with no risk alleles is even larger. For each plot, the genotype effect sizes reflect the association between BMI and the specified variable for each genotype.

Gender-adjusted linear models were also used to test the association between the *FTO* rs1421085–rs17817449 haplotype and each lifestyle and psychological health variable, as well as the effect of the interaction between this haplotype and each lifestyle and psychological health variable on BMI. Plots depicting significant additive allelic interactions were drawn. The haplotype analyses determine whether a statistical significant difference exists between the slopes of all haplotype groups. The slopes that are significantly different from each other on each plot are indicated in the results section.

Data were analyzed using statistical program R [61] and R packages genetics and haplo.stats. All results corresponding to a *p*-value of <0.05 were described as statistically significant. It should be noted that multiple comparisons, as performed in this study, are recognized as a common statistical approach in genetic association studies [62]. Furthermore, it has been suggested that adjustments, such as Bonferroni, are too strict and may result in missing significant associations when performing multiple association tests in one group of individuals [63]. Therefore, we did not adjust for multiple testing but rather considered the results of all association tests while interpreting each significant *p*-value with caution, taking into account the plausibility of the finding as recommended by Perneger [64]. Some results may thus be considered hypotheses generating, whereas the replication of previously reported results and functional explanation of the association between the polymorphism and phenotype may verify plausibility of such results [62].

## 3. Results

### 3.1. Socio-Demographic and Weight Profile of the Cross-Sectional Sample

The subjects had a mean ± SD age of 32.9 ± 4.4 years, weight of 99.9 ± 20.1 kg, height of 1.68 ± 0.08 m and BMI of 35.2 ± 6.6 kg/m^2^. In this sample 21.8% of subjects were overweight (BMI of 27–29.9 kg/m^2^), 33.1% were obese class I (BMI of 30–34.9 kg/m^2^), 24.1% were obese class II (BMI of 35–39.9 kg/m^2^) and 21.0% were obese class III (BMI ≥ 40 kg/m^2^). Most were female, married, Afrikaans speaking, had a tertiary qualification, and lived with their partners and children (Table 2). There were no significant differences in mean BMI between the response categories of the socio-demographic variables.

**Table 2:** Socio-demographic profile and genotype, allele and haplotype frequencies of the *FTO* rs1421085 and rs17817449 polymorphisms.

Socio-Demographic Variables and Polymorphisms	*n*	%	BMI (kg/m^2^) Mean ± SD	*p*-Value
Gender				
Female	112	84.2	35.3 ± 6.9	0.818
Male	21	15.8	34.9 ± 4.5	
Marital status				
Married/living together	86	64.7	35.1 ± 6.3	0.703
Unmarried (including separated/divorced)	47	35.3	35.5 ± 7.1	
Home language				
Afrikaans	120	90.2	35.2 ± 6.5	0.729
English	13	9.8	35.8 ± 7.2	
Level of education				
Completed Grade 10 or Matric	43	32.3	35.1 ± 7.3	0.887
Tertiary qualification	90	67.7	35.3 ± 6.2	
Living				
alone	22	16.5	37.4 ± 8.0	0.156
with friends/parents	20	15.0	33.7 ± 6.0	
with a partner	30	22.6	33.8 ± 5.6	
with a partner and child (ren)	61	45.9	35.7 ± 6.5	
*FTO* rs1421085 polymorphism				
Genotype frequencies: TT	21	20.8	34.5 ± 7.3	0.1566
TC	55	54.5	36.6 ± 7.0	
CC	25	24.7	33.6 ± 5.4	
Allele frequencies: Risk allele: C		52.0		0.5831
*FTO* rs17817449 polymorphism				
Genotype frequencies: TT	31	29.5	35.9 ± 7.0	0.7814
TG	32	30.5	34.9 ± 6.8	
GG	42	40.0	36.0 ± 7.2	
Allele frequencies: Risk allele: G		55.2		0.9178
*FTO* rs1421085-rs17817449 haplotype frequencies				
Haplotypes: C-G	*	27.3	*	0.6010
C-T		24.7		
T-G		27.9		
T-T		20.0		

* Where haplotype allocation was uncertain (*i.e.*, when a subject is heterozygous T-C and T-G for both polymorphisms, it cannot be established whether the haplotype for the individual is for example T-T or C-G), different pairs of haplotypes, with probabilities of being the true haplotype were inferred for individuals and thus cannot be counted.

### 3.2. Genotype and Allele Frequencies

The frequencies of the homozygous GG genotype (*n* = 42, 40.0%) of the *FTO* rs17817449 polymorphism and the heterozygous TC genotype of the *FTO* rs1421085 polymorphism were the most prevalent (Table 2). The frequencies of the risk alleles of both *FTO* polymorphisms were higher than the respective non-risk T-alleles. The genotype frequencies of the *FTO* rs1421085 polymorphism were in HWE (*p* = 0.4284), while the genotype frequencies of the *FTO* rs17817449 polymorphisms were not in HWE (*p* = 0.0001). The frequency of the non-risk T-T haplotype of the *FTO* rs1421085–rs17817449 haplotype was the lowest in this sample (Table 2). No associations between BMI and genotype groups, additive allelic scores or haplotypes were found.

### 3.3. Dietary Intake of Indicator Food Groups

Subjects with the homozygous GG genotype of the *FTO* rs17817449 polymorphism had a 1.74 and 0.67 times higher intake of high-fat foods and refined starches, respectively, than subjects with one or no risk alleles (Table 3). No significant associations were found between the *FTO* polymorphisms and energy dense snacks or drinks consumed.

**Table 3:** Significant associations between genotype groups of *FTO* rs1421085 and rs17817449 polymorphisms and lifestyle/psychological health variables.

Lifestyle/Psychological Health Variable	Polymorphisms	Genotypes	*n* *	Mean ± SD	Effect Size ± SE	Model	*p*-Value
**Food groups**				**Frequency of intake/day**			
High fat foods	*FTO* rs17817449	T-allele carriers	59	4.9 ± 3.6	1.74 ± 0.87	Recessive G	0.0494
		GG	38	6.6 ± 4.9			
Refined starches	*FTO* rs17817449	T-allele carriers	59	0.94 ± 1.36	0.67 ± 0.30	Recessive G	0.0287
		GG	38	1.59 ± 1.55			
**TFEQ**				**Questionnaire score**			
Perceived hunger	*FTO* rs1421085	TT	20	5.9 ± 3.4	1.43 ± 0.52	Additive C	0.0072
		TC	54	7.3 ± 3.0			
		CC	23	8.8 ± 3.9			
Hunger: Internal locus of control	*FTO* rs1421085	TT	20	2.2 ± 1.7	0.84 ± 0.28	Additive C	0.0038
		TC	54	2.8 ± 1.8			
		CC	23	3.9 ± 2.0			
Emotional disinhibition	*FTO* rs1421085	TT	20	2.0 ± 1.3	0.58 ± 0.26	Dominant C	0.0281
		C-allele carriers	77	2.4 ± 1.1			
Restraint scale:	*FTO* rs1421085	TT	20	1.7 ± 1.6	0.89 ± 0.43	Dominant C	0.0392
Flexible control		C-allele carriers	77	2.5 ± 1.7			
**BDI**	*FTO* rs17817449	T-allele carriers	61	12.7 ± 9.4	4.58 ± 2.02	Recessive G	0.0256
		GG	40	17.6 ± 10.7			

BDI = Beck depression inventory, RSES = Rosenberg self-esteem scale. * *n* vary due to missing values. Risk allele for *FTO* rs1421085 = C-allele and for *FTO* rs17817449 = G-allele. Non-significant differences not included in table.

### 3.4. Eating Behavior

The association between the *FTO* rs1421085 polymorphism and perceived hunger was significant for the recessive, dominant, genotype, and additive allelic models, with the latter being the best fit. It was found that the perceived hunger score of subjects with one risk allele was 1.43 times higher than the score of subjects with no risk alleles, while those with two risk alleles had a 2.86 times higher score (Table 3). Similarly, subjects with two risk alleles of the *FTO* rs1421085 polymorphism had the highest internal locus of control for hunger scores compared to those with one or no C-alleles. Furthermore, the risk C-allele carriers also had higher emotional disinhibition and flexible control scores compared to the TT homozygotes (Table 3).

**Figure 1:**
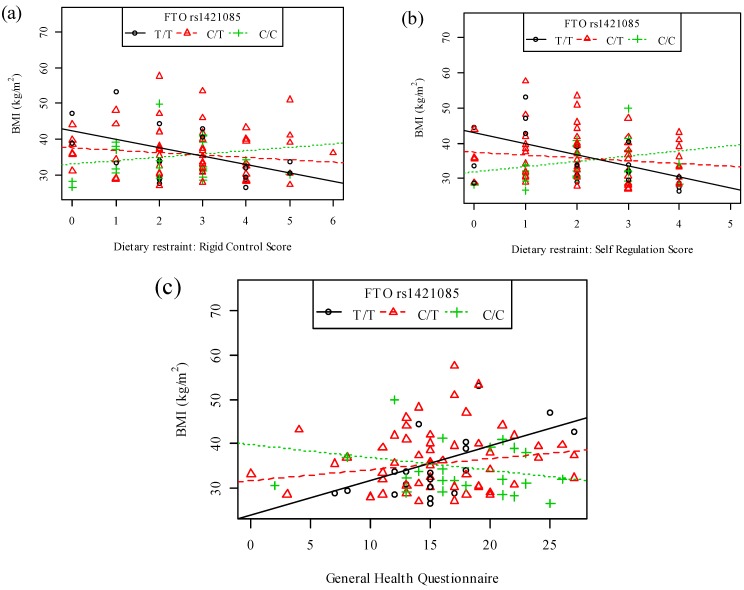
Interactions between the *FTO* rs1421085 polymorphism and eating behavior or psychological health on BMI. Plots of BMI against (**a**) rigid control score (*p* = 0.0290); (**b**) attitude to self-regulation score (*p* = 0.0095) and (**c**) General Health Questionnaire score (*p* = 0.0085). Symbols represent all individual observed values for males and females and regression lines show the expected relationships for a female, from the additive allelic model, for each genotype of the *FTO* rs1421085 polymorphism.

The rate of change in BMI in response to a change in the rigid control (allelic effect size =1.67, *p* = 0.0290, Figure 1a) and self-regulation (allelic effect size = 2.29, *p* = 0.0095, Figure 1b) subscale scores differed significantly between the genotype groups of the *FTO* rs1421085 polymorphism. The addition of one risk C-allele results in a 1.67-unit or 2.29-unit **increase** in the slope (rate of change) of BMI for each one-unit increase in the rigid control (Figure 1a) or self-regulation (Figure 1b) score, respectively, when compared to subjects with no risk alleles. Therefore, the addition of two risk alleles would result in a 3.34-unit or 4.58-unit increase in the slope of BMI for each one-unit increase in the rigid control or self-regulation scores, respectively. The most pronounced decrease in BMI in response to an increase in the rigid control (genotype effect size = −2.37, *p* = 0.0078 for the TT regression line on Figure 1a) or attitude to self-regulation scores (genotype effect size = −3.11; *p* = 0.0078 for the TT regression line on Figure 1a) was observed for the non-risk TT homozygotes. Thus, each one-unit increase in the rigid control or self-regulation score of TT homozygotes results in a 2.37-unit or 3.11-unit decrease in BMI, respectively. The unit change in BMI in response to an increase in the rigid control score of the CT (−2.37 + 1.67 = −0.70 unit) and CC (−2.37 + 3.34 = 0.97 unit) genotypes was smaller than the TT homozygotes and non-significant. Similarly, smaller non-significant changes in BMI in response to an increase in the self-regulation score (CT: −3.11 plus 2.29 = −0.82 unit and CC: −3.11 plus 4.58 = 1.47 unit) were found.

**Figure 2:**
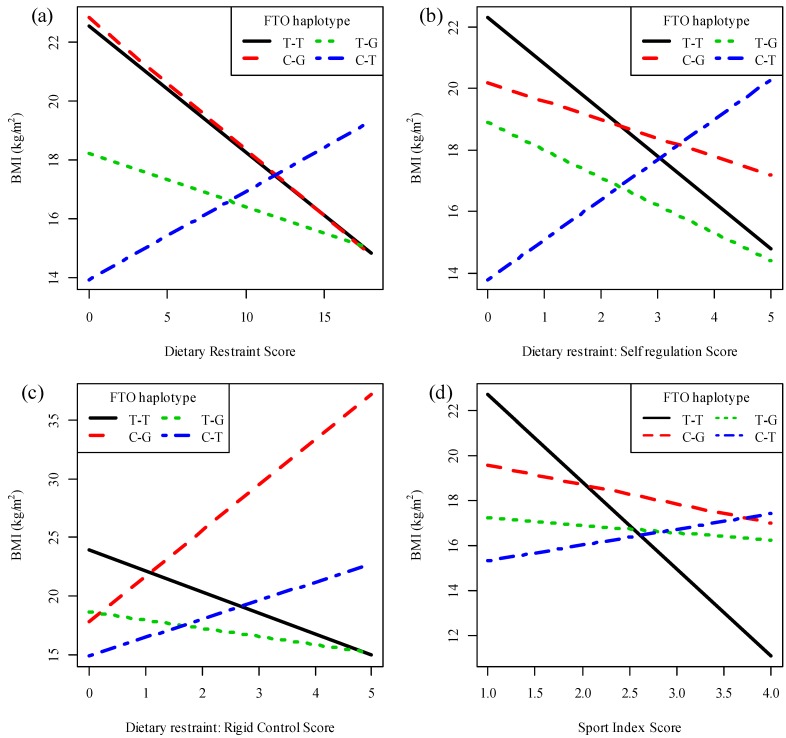
Interactions between the fat mass and obesity-associated (*FTO*) rs1421085-rs17817449 haplotype and eating behavior or physical activity on BMI. Plots of BMI against (**a**) dietary restraint; (**b**) self-regulation; (**c**) rigid control and (**d**) sport index scores. Regression lines show the expected relationships, for a woman, from the additive haplotype model, for each *FTO* rs1421085-rs17817449 haplotype.

Haplotype analyses revealed that the rate of change in BMI in response to a change in the dietary restraint (effect size = 0.74, SE = 0.35, *p* = 0.0389, Figure 2a) and self-regulation (effect size = 2.7, SE = 1.21, *p* = 0.0273, Figure 2b) scores differed significantly between the T-T and C-T haplotypes of the two *FTO* polymorphisms. Although it is evident from Figure 2a,b that an increase in dietary restraint or attitude to self-regulation was associated with a decrease in BMI for each T-T haplotype and an increase in BMI for each C-T haplotype, these individual associations were not statistically significant.

The rate of change in BMI in response to a change in rigid control differed significantly between the T-T and C-T (effect size = 2.25, SE = 0.95, *p* = 0.0190) as well as the T-T and C-G (effect size = 2.31, SE = 1.03, *p* = 0.0268) haplotypes of the two *FTO* polymorphisms (Figure 2c). For each T-T haplotype, a one-unit increase in the rigid control score results in a decrease in BMI of 1.79 kg/m^2^ (effect size = 1.79, *p* = 0.010). The change in BMI in response to a change in the rigid control score was not significant for any of the other haplotypes.

### 3.5. Physical Activity

No associations were found between the *FTO* rs1421085 or rs17817449 polymorphisms and physical activity levels (results not reported). However, the rate of change in BMI in response to a change in the sports index score differed significantly between the T-T and C-T (effect size = 4.57, SE = 1.95, *p* = 0.0212) as well as between the T-T and T-G (effect size = 3.53, SE = 1.75, *p* = 0.0469) haplotypes of the two *FTO* polymorphisms (Figure 2d). For each T-T haplotype, a one-unit increase in the sport index score results in a decrease in BMI of 3.87 kg/m^2^ (effect size = 3.87, *p* = 0.004). The change in BMI in response to a change in the sport index score was not significant for any of the other haplotypes.

### 3.6. Psychological Health

No associations were found between the genotypes of the *FTO* polymorphisms and the RSES or GHQ scores. Significantly higher BDI scores were found in the GG homozygotes of the *FTO* rs17817449 polymorphism (Table 3).

The rate of change in BMI in response to a change in the GHQ score differed significantly between the genotype groups of the *FTO* rs1421085 polymorphism (allelic effect size = −0.53, *p* = 0.0085, Figure 1c). The addition of one risk C-allele results in a 0.53-unit **decrease** in the slope of BMI for each one-unit increase in the GHQ score when compared to TT homozygotes with no risk alleles. Therefore, the addition of two risk alleles would result in a 1.06-unit decrease in the slope of BMI for each one-unit increase in the GHQ score. The most pronounced increase in BMI in response to an increase in GHQ score was observed for the non-risk TT homozygotes (genotype effect size = 0.78, *p* = 0.0026). Thus, each one-unit increase in the GHQ score of TT homozygotes results in a 0.78-unit increase in BMI. The unit change in BMI in response to an increase in the GHQ scores of the CT (0.78 − 0.53 = 0.25 unit) and CC (0.78 − 1.06 = −0.28 unit) genotypes was smaller than the TT homozygotes and non-significant.

## 4. Discussion

The main findings of this study show associations between the *FTO* rs1421085 and rs17817449 polymorphisms and indicators of eating behavior, dietary intake, physical activity, and psychological health. Furthermore, our findings show that the interactions between these variables and the *FTO* polymorphisms may be associated with the BMI of overweight/obese Caucasian adults. These results support the proposed involvement of *FTO* polymorphisms in regulation of energy homeostasis [32,36].

Our results indicate that subjects with the risk GG genotype of the *FTO* rs17817449 polymorphism had a higher intake of high fat foods and refined starches. Furthermore, subjects with the risk C-allele of the *FTO* rs1421085 polymorphism were characterized by a higher perceived hunger score, which reflects a higher susceptibility to general subjective feelings and perceptions of hunger that may result in higher food intake [48]. Subscale analysis of the hunger score revealed that the perceived hunger of C-allele carriers was internally interpreted and regulated (higher internal locus for hunger score). This implies that these subjects perceived themselves to be hungry more often and finding themselves “just having to eat something at any time”, thus possibly resulting in eating more frequently during the day [50]. The C-allele carriers were also characterized by a higher emotional disinhibition score, which reflects disinhibition that arises as a result of emotional feelings such as loneliness, anxiousness or feeling depressed [50]. These significant associations from our study point to the possibility that subjects with one or more risk alleles of the *FTO* rs1421085 or rs17817449 polymorphisms may have poorer eating behaviors and a higher intake of high fat foods or refined starches. However, it must be noted that the fact that C-allele carriers had a higher flexible control score (subscale score of dietary restraint), may not support this notion. A higher flexible control score reflects better eating behavior, namely the occasional intake of sweets, treats, and high fat foods, but in limited amounts. This approach reflects a more consistent restraint that is conducive to effective weight management strategies [49].

To our knowledge this is the first study to report on the associations between the *FTO* rs1421085 or rs17817449 polymorphisms and TFEQ subscales. This is also the first study to report on the *FTO* rs17817449 polymorphism and food intake, as well as the TFEQ scales. In the only publication that could be traced that investigated the associations between the *FTO* rs1421085 polymorphism and the TFEQ scales, snacking or eating large quantities of food, no associations were found [38]. This is in contrast to the association we find between this polymorphism and perceived hunger. It can be argued that our results are in line with those of McCaffery *et al.* [37], who reported a higher daily intake of energy, percentage fat contribution to total energy, number of meals and snacks, as well as servings of fats, oils, and sweets by the risk allele carriers of the *FTO* rs1421085 polymorphism. Furthermore, support for possible associations between dietary intake and eating behaviors and the *FTO* gene comes from research on polymorphisms located in the same LD block as the rs1421085 and rs17817449 polymorphisms. In children, the high risk genotype or allele of the *FTO* rs9939609 polymorphism was associated with impaired ability to respond to satiety cues [65], a higher intake of highly palatable foods [36], a higher energy intake [29], more frequent “loss of control” eating episodes, and a higher intake of high fat foods at a lunch buffet [35]. However, some studies were unable to replicate these results in children or adolescents [33,66]. In Caucasian adults, a greater number of meals and snacks per day [37] and a higher energy intake from fat [67] and a higher energy intake were associated with the risk alleles of the *FTO* rs9939609 [32] and rs8050136 polymorphisms [34]. Most of the evidence at this stage thus seems to support our findings that the risk allele carriers of the *FTO* polymorphisms may have poorer eating behaviors. It could thus be argued that our finding that the risk genotype has higher flexible control is possibly a false positive.

Our modeling results illustrate that interactions between *FTO* genotypes or haplotypes investigated in this study and eating behavior may be associated with the BMI of subjects. For instance, the results indicate that subjects with the non-risk TT genotype of the *FTO* rs1421085 polymorphism may benefit from improving their attitude to self-regulation score (subscale score of dietary restraint) when attempting weight reduction, while the same beneficial effect may not be experienced by the risk allele carriers. It was evident that an increase in the self-regulation score of one-unit was associated with a significant 3.11 kg/m^2^ decrease in BMI. An increase in attitude to self-regulation score denotes increased dietary restraint and reflects a subject’s general view on eating and the importance of dieting, more specifically consideration of the energy content of food and eating specific foods at specific times for weight control [50]. Conflicting results regarding the association between dietary restraint and BMI have been reported. However, the potential benefit from increasing dietary restraint during weight reduction is supported by the fact that a lower restraint score has been associated with higher body weight [68,69] and that decreases in dietary restraint over time were associated with concurrent weight gain [70]. Furthermore, an increase in dietary restraint during weight loss was associated with a greater weight loss [49,71] and was found to be a significant predictor of weight maintenance over two years [72]. It is thus not surprising that Hill *et al.* [73] argue in a review article that chronic dietary restraint is necessary to be an effective weight manager in the current obesogenic environment. Our subscale analysis of dietary restraint further indicates that a modeled increase in the rigid control score was associated with the most pronounced decrease in BMI of subjects with the non-risk TT genotype of the *FTO* rs1421085 polymorphism and a significant decrease in the BMI of subjects with a T-T haplotype. Rigid control specifically relates to the complete avoidance of “fattening foods”, sweets and/or treats, with individuals employing an all-or-nothing approach to dieting. Over the short-term, a high rigid control may be associated with decreases in BMI, which may explain our results. However, high rigid control is not recommended for effective weight management purposes, as frequent lapses or total relapse may occur, which explains the higher BMIs, energy intakes, and less success with weight loss in individuals with a higher rigid control [49,74]. It should be noted that the other significant interactions found between the *FTO* haplotype, BMI, and dietary restraint, as well as attitude to self-regulation scores, cannot be further interpreted as no individual haplotype was significantly associated with BMI and eating behavior changes.

A role for *FTO* in the regulation of energy expenditure has also been proposed and investigated. Although no studies could be traced that investigated the association between physical activity and the *FTO* rs1421085 or rs17817449 polymorphisms, the association with the *FTO* rs9939609 polymorphism has been examined. Some studies have failed to show any association with indicators of physical activity levels and energy expenditure [31,32,33,36,66]. This is in line with our results that show no association between the genotype groups of the *FTO* rs1421085 or rs17817449 polymorphisms and self-reported physical activity levels at work, during leisure-time or sport. In contrast, an association between the risk alleles of the *FTO* rs9939609 polymorphism and increased energy expenditure has been reported by Cecil *et al.* [29] and Do *et al.* [30]. The possibility of an association between BMI, the *FTO* gene, and physical activity levels is also supported by the results from a meta-analysis indicating that adults who were physically inactive and carriers of the risk allele of the *FTO* rs9939609 polymorphism had a higher obesity risk than their physically active counterparts [75]. In line with the latter results, our haplotype analysis indicates that each one-unit increase in physical activity levels (sport index) was associated with a significant 3.87 kg/m^2^ decrease in BMI for each non-risk T-T haplotype. This implies that subjects with no risk alleles of the two *FTO* polymorphisms may benefit the most from increasing their physical activity levels when attempting weight loss. In practical terms, a one-unit increase in the sport index score of a physically inactive subject can easily be achieved by practicing a low-level activity such as walking for 1–2 h per week. Subjects who already walk 1–2 h per week should increase this to 3–4 h per week and additionally practice a medium level activity such as swimming, jogging, aerobics or cycling for at least one hour per week. Instead, walking time can also be fully substituted for these medium level activities (3–4 h per week) to achieve the one-unit increase in sport index score (all recommendations were calculated using the equations for calculating the sport index score by Baecke *et al.* [44]).

It is known that various mental and personality disorders such as mania, anxiety, and depression contribute to the hedonic aspects of overeating and may predict the development of obesity [76,77]. As *FTO* is expressed throughout the brain it may be associated with psychological behaviors such as depression or psychological distress and consequent BMI of individuals. Although no reports could be traced on the association between psychological well-being and the *FTO* rs1421085 or rs17817449 polymorphisms, associations with other *FTO* polymorphisms have recently been investigated [78,79]. For instance, associations between BMI, depression, and 10 polymorphisms in the *FTO* gene have been reported by Rivera *et al.* [78]. We also found a significant association between the *FTO* rs1421085 polymorphism, BMI, and general psychological well-being. It was specifically found that each one-unit increase in GHQ-score was associated with the most pronounced 0.78 kg/m^2^ increase in the BMI of the non-risk TT subjects of the *FTO* rs1421085 polymorphism. The GHQ score is calculated from 30 questions and each question may contribute one-unit to the total score if the answer reflects the psychological distress option. Therefore, worsening of general psychological well-being may readily result in a few units increase in the GHQ score and consequently a significant increase in BMI. Our results further suggest that the at-risk GG subjects of the *FTO* rs17817449 polymorphism may experience more depressive symptoms (as measured by the BDI). In contrast to our results, Samaan *et al.* [79] reported that the risk A-allele of *FTO* rs9939609 was associated with a lower risk of depression [79]. As a limited number of reports have investigated these associations, more research is clearly necessary to understand the association between psychological health, BMI, and the *FTO* gene.

This study is limited by the fact that most variables were self-reported, including physical activity, dietary intake, eating behavior, and psychological health, which may not necessarily reflect the truth. We aimed to improve the validity of these outcome measures by using instruments validated in similar populations and checking the internal consistency reliability. Although content and face validity of the food lists included in the FFQ were ensured, validation of the daily frequency of intake of items in the four indicator food groups against another method, e.g., food records, is recommended. It is important to note that the results of this study pertain to overweight/obese individuals only. This is reflected by the genotype and allele frequencies of the two polymorphisms investigated in this sample, which were in line with those previously reported for obese Caucasian populations [7,80,81]. Although the sample did not include normal weight participants, the BMI range of included participants (27.0 kg/m^2^ to 57.6 kg/m^2^) supported the potential for investigation of associations between BMI, genotype and lifestyle, eating behavior and psychological variables. The deviation found in HWE for the *FTO* rs17817449 polymorphism also needs to be considered in the interpretation of the results for this polymorphism. As genotyping errors can be excluded, we suggest that the fact that the sample is not representative of a general population (did not include subjects with a BMI < 27) could explain the HWE finding. As most obesity related polymorphisms are expected to have a small effect on obesity development or weight loss outcomes, the sample size restricted statistical power of analyses. This may have resulted in non-detection of possible associations.

## 5. Conclusions

The risk alleles of the *FTO* rs1421085 and rs17817449 polymorphisms may be associated with poorer eating behaviors (reflected by higher hunger, internal locus for hunger, and emotional disinhibition scores), a higher intake of high fat foods and refined starches and more depressive symptoms in treatment seeking overweight/obese Caucasian adults. Furthermore, our modeled results indicate that the interactions between the *FTO* polymorphisms or haplotypes and eating behavior, psychological health and physical activity levels may be associated with BMI. A higher rigid control and attitude to self-regulation score may decrease the BMI of the non-risk homozygotes of *FTO* rs1421085, while an increase in psychological distress (increased GHQ score) may increase their BMI. Furthermore, increased sport participation may result in a lower BMI in subjects with the non-risk haplotype. These results may explain current findings that the risk alleles of both *FTO* polymorphisms have consistently been associated with a higher BMI or obesity prevalence [7,9,30,38,80,81]. We recommend that the novel associations found in the current study should be viewed as “hypotheses generating” and that they should be replicated and further investigated to establish the clinical significance for implementation as part of weight management interventions.

## References

[1] World Health Organization (2003). Diet, Nutrition and the Prevention of Chronic Diseases. Report for a Joint Who/Fao Expert Consultation.

[2] Obesity: Situation and Trends. http://www.who.int/gho/ncd/risk_factors/obesity_text/en/index.html.

[3] Marti A., Martinez-González M.A., Martinez J.A. (2008). Interaction between genes and lifestyle factors on obesity. Proc. Nutr. Soc..

[4] Romao I., Roth J. (2008). Genetic and environmental interactions in obesity and type 2 diabetes. J. Am. Diet. Assoc..

[5] Larder R., Cheung M.K., Tung Y.C., Yeo G.S., Coll A.P. (2011). Where to go with FTO?. Trends Endocrinol. Metab..

[6] Speliotes E.K., Willer C.J., Berndt S.I., Monda K.L., Thorleifsson G., Jackson A.U., Lango Allen H., Lindgren C.M., Luan J., Mägi R. (2010). Association analyses of 249,796 individuals reveal 18 new loci associated with body mass index. Nat. Genet..

[7] Dina C., Meyre D., Gallina S., Durand E., Körner A., Jacobson P., Carlsson L.M., Kiess W., Vatin V., Lecoeur C. (2007). Variation in *FTO* contributes to childhood obesity and severe adult obesity. Nat. Genet..

[8] Frayling T.M., Timpson N.J., Weedon M.N., Zeggini E., Freathy R.M., Lindgren C.M., Perry J.R., Elliott K.S., Lango H., Rayner N.W. (2007). A common variant in the *FTO* gene is associated with body mass index and predisposes to childhood and adult obesity. Science.

[9] Scuteri A., Sanna S., Chen W.M., Uda M., Albai G., Strait J., Najjar S., Nagaraja R., Orru M., Usala G. (2007). Genome-wide association scan shows genetic variants in the *FTO* gene are associated with obesity-related traits. PLoS Genet..

[10] Frayling T.M., Ong K. (2011). Piecing together the *FTO* jigsaw. Genome Biol..

[11] Gerken T., Girard C.A., Tung Y.C., Webby C.J., Saudek V., Hewitson K.S., Yeo G.S., McDonough M.A., Cunliffe S., McNeill L.A. (2007). The obesity-associated *FTO* gene encodes a 2-oxoglutarate-dependent nucleic acid demethylase. Science.

[12] Sanchez-Pulido L., Andrade-Navarro M.A. (2007). The *FTO* (fat mass and obesity associated) gene codes for a novel member of the non-heme dioxygenase superfamily. BMC Biochem..

[13] Han Z., Niu T., Chang J., Lei X., Zhao M., Wang Q., Cheng W., Wang J., Feng Y., Chai J. (2010). Crystal structure of the *FTO* protein reveals basis for its substrate specificity. Nature.

[14] Fawcett K.A., Barroso I. (2010). The genetics of obesity: FTO leads the way. Trends Genet..

[15] Jia G., Yang C., Yang S., Jian X., Yi C., Zhou Z., He C. (2008). Oxidative demethylation of 3-methylthymine and 3-methyluracil in single-stranded DNA and RNA by mouse and human *FTO*. FEBS Lett..

[16] Jia G., Fu Y., Zhao X., Dai Q., Zheng G., Yang Y., Yi C., Lindahl T., Pan T., Yang YG., He C. (2011). N6-methyladenosine in nuclear RNA is a major substrate of the obesity-associated *FTO*. Chem. Biol..

[17] Fredriksson R., Hägglund M., Olszewski P.K., Stephansson O., Jacobsson J.A., Olszewska A.M., Levine A.S., Lindblom J., Schiöth H.B. (2008). The obesity gene, *FTO*, is of ancient origin, up-regulated during food deprivation and expressed in neurons of feeding-related nuclei of the brain. Endocrinology.

[18] Stratigopoulos G., Padilla S.L., LeDuc C.A., Watson E., Hattersley A.T., McCarthy M.I., Zeltser L.M., Chung W.K., Leibel R.L. (2008). Regulation of *Fto/Ftm* gene expression in mice and humans. Am. J. Physiol. Regul. Integr. Comp. Physiol..

[19] Tung Y.C., Ayuso E., Shan X., Bosch F., O’Rahilly S., Coll A.P., Yeo G.S. (2010). Hypothalamic-specific manipulation of *Fto*, the ortholog of the human obesity gene *FTO*, affects food intake in rats. PLoS One.

[20] Poritsanos N.J., Lew P.S., Fischer J., Mobbs C.V., Nagy J.I., Wong D., Rüther U., Mizuno T.M. (2011). Impaired hypothalamic *Fto* expression in response to fasting and glucose in obese mice. Nutr. Diabetes.

[21] Wåhlén K., Sjölin E., Hoffstedt J. (2008). The common rs9939609 gene variant of the fat mass- and obesity-associated gene *FT*O is related to fat cell lipolysis. J. Lipid Res..

[22] Zabena C., González-Sánchez J.L., Martínez-Larrad M.T., Torres-García A., Alvarez-Fernández-Represa J., Corbatón-Anchuelo A., Pérez-Barba M., Serrano-Ríos M. (2009). The *FTO* obesity gene. Genotyping and gene expression analysis in morbidly obese patients. Obes. Surg..

[23] Fischer J., Koch L., Emmerling C., Vierkotten J., Peters T., Brüning J.C., Rüther U. (2009). Inactivation of the *Fto* gene protects from obesity. Nature.

[24] Cheung M.K., Gulati P., O’Rahilly S., Yeo G.S. (2013). *FTO* expression is regulated by availability of essential amino acids. Int. J. Obes..

[25] Pitman R.T., Fong J.T., Billman P., Puri N. (2012). Knockdown of the fat mass and obesity gene disrupts cellular energy balance in a cell-type specific manner. PLoS One.

[26] Church C., Moir L., McMurray F., Girard C., Banks G.T., Teboul L., Wells S., Brüning J.C., Nolan P.M., Ashcroft F.M. (2010). Overexpression of *Fto* leads to increased food intake and results in obesity. Nat. Genet..

[27] McMurray F., Church C.D., Larder R., Nicholson G., Wells S., Teboul L., Tung Y.C., Rimmington D., Bosch F., Jimenez V. (2013). Adult onset global loss of the *Fto* gene alters body composition and metabolism in the mouse. PLoS Genet..

[28] Gao X., Shin Y.H., Li M., Wang F., Tong Q., Zhang P. (2010). The fat mass and obesity associated gene *FTO* functions in the brain to regulate postnatal growth in mice. PLoS One.

[29] Cecil J.E., Tavendale R., Watt P., Hetherington M.M., Palmer C.N. (2008). An obesity-associated *FTO* gene variant and increased energy intake in children. N. Engl. J. Med..

[30] Do R., Bailey S.D., Desbiens K., Belisle A., Montpetit A., Bouchard C., Pérusse L., Vohl M.C., Engert J.C. (2008). Genetic variants of *FTO* influence adiposity, insulin sensitivity, leptin levels, and resting metabolic rate in the Quebec Family Study. Diabetes.

[31] Berentzen T., Kring S.I., Holst C., Zimmermann E., Jess T., Hansen T., Pedersen O., Toubro S., Astrup A., Sørensen T.I. (2008). Lack of association of fatness-related *FTO* gene variants with energy expenditure or physical activity. J. Clin. Endocrinol. Metab..

[32] Speakman J.R., Rance K.A., Johnstone A.M. (2008). Polymorphisms of the *FTO* gene are associated with variation in energy intake, but not energy expenditure. Obesity.

[33] Hakanen M., Raitakari O.T., Lehtimäki T., Peltonen N., Pahkala K., Sillanmäki L., Lagström H., Viikari J., Simell O., Rönnemaa T. (2009). *FTO* genotype is associated with body mass index after the age of seven years but not with energy intake or leisure-time physical activity. J. Clin. Endocrinol. Metab..

[34] Haupt A., Thamer C., Staiger H., Tschritter O., Kirchhoff K., Machicao F., Häring H.U., Stefan N., Fritsche A. (2009). Variation in the *FTO* gene influences food intake but not energy expenditure. Exp. Clin. Endocrinol. Diabetes.

[35] Tanofsky-Kraff M., Han J.C., Anandalingam K., Shomaker L.B., Columbo K.M., Wolkoff L.E., Kozlosky M., Elliott C., Ranzenhofer L.M., Roza C.A. (2009). The *FTO* gene rs9939609 obesity-risk allele and loss of control over eating. Am. J. Clin. Nutr..

[36] Wardle J., Llewellyn C., Sanderson S., Plomin R. (2009). The *FTO* gene and measured food intake in children. Int. J. Obes..

[37] McCaffery J.M., Papandonatos G.D., Peter I., Huggins G.S., Raynor H.A., Delahanty L.M., Cheskin L.J., Balasubramanyam A., Wagenknecht L.E., Wing R.R. et al. (2012). Obesity susceptibility loci and dietary intake in the Look AHEAD Trial. Am. J. Clin. Nutr..

[38] Stutzmann F., Cauchi S., Durand E., Calvacanti-Proença C., Pigeyre M., Hartikainen A.L., Sovio U., Tichet J., Marre M., Weill J. (2009). Common genetic variation near MC4R is associated with eating behavior patterns in European populations. Int. J. Obes..

[39] Miller S.A., Dykes D.D., Polesky H.F. (1988). A simple salting out procedure for extracting DNA from human nucleated cells. Nucleic Acids Res..

[40] Norton K., Olds T. (1996). Anthropometrica: A Textbook of Body Measurement for Sports and Health Courses.

[41] Albanes D., Conway J.M., Taylor P.R., Moe P.W., Judd J. (1990). Validation and comparison of eight physical activity questionnaires. Epidemiology.

[42] Miller D.J., Freedson P.S., Kline G.M. (1994). Comparison of activity levels using the Caltrac accelerometer and five questionnaires. Med. Sci. Sports Exerc..

[43] Richardson M.T., Ainsworth B.E., Wu H., Jacobs D.R., Leon A.S. (1995). Ability of the atherosclerosis risk in communities (ARIC)/Baecke questionnaire to assess leisure-time physical activity. Int. J. Epidemiol..

[44] Baecke J.A.H., Burema J., Frijters J.E.R. (1982). A short questionnaire for the measurement of habitual physical activity in epidemiological studies. Am. J. Clin. Nutr..

[45] Steyn N.P., Senekal M. (2005). A Guide for the Use of the Dietary Assessment and Education Kit (DAEK).

[46] Laessle R.G., Tuschl R.J., Kotthaus B.C., Pirke K.M. (1989). A comparison of the validity of three scales for the assessment of dietary restraint. J. Abnorm. Psychol..

[47] Provencher V., Drapeau V., Tremblay A., Després J.P., Lemieux S. (2003). Eating behaviors and indexes of body composition in men and women from the Québec Family Study. Obes. Res..

[48] Stunkard A.J., Messick S. (1985). The three-factor eating questionnaire to measure dietary restraint, disinhibition and hunger. J. Psychosom. Res..

[49] Westenhoefer J., Stunkard A.J., Pudel V. (1999). Validation of the flexible and rigid control dimensions of dietary restraint. Int. J. Eat. Disord..

[50] Bond M.J., McDowell A.J., Wilkinson J.Y. (2001). The measurement of dietary restraint, disinhibition and hunger: An examination of the factor structure of the Three Factor Eating Questionnaire (TFEQ). Int. J. Obes..

[51] Bas M., Donmez S. (2009). Self-efficacy and restrained eating in relation to weight loss among overweight men and women in Turkey. Appetite.

[52] Chaput J.P., Leblanc C., Perusse L., Despres J.P., Bouchard C., Tremblay A. (2009). Risk factors for adult overweight and obesity in the Quebec Family Study: Have we been barking up the wrong tree?. Obesity.

[53] Beck A.T., Steer R.A. (1987). Manual for the Beck Depression Inventory.

[54] Beck A.T., Steer R.A., Brown G.K. (1996). BDI-II Manual.

[55] Goldberg D.P. (1972). The Detection of Psychiatric Illness by Questionnaire.

[56] Goldberg D.P., Rickels K., Downing R., Hesbacher P. (1976). A comparison of two psychiatric screening tests. Br. J. Psychiatry.

[57] Banks M.H. (1983). Validation of the General Health Questionnaire in a young community sample. Psychol. Med..

[58] Lorr M., Wunderlich R.A. (1986). Two objective measures of self-esteem. J. Pers. Assess..

[59] Reynolds W.M. (1988). Measurement of academic self-concept in college students. J. Pers. Assess..

[60] Rosenberg M. (1965). Society and the Adolescent Self-Image.

[61] R: A Language and Environment for Statistical Computing. R Foundation for Statistical Computing, Vienna, Austria. http://www.R-project.org/.

[62] Andersson U., McKean-Cowdin R., Hjalmars U., Malmer B. (2009). Genetic variants in association studies-review of strengths and weaknesses in study design and current knowledge of impact on cancer risk. Acta Oncol..

[63] Nyholt D.R. (2004). A simple correction for multiple testing for single-nucleotide polymorphisms in linkage disequilibrium with each other. Am. J. Hum. Genet..

[64] Perneger T.V. (1998). What’s wrong with Bonferroni adjustments. BMJ.

[65] Wardle J., Carnell S., Haworth C.M., Farooqi I.S., O’Rahilly S., Plomin R. (2008). Obesity associated genetic variation in *FTO* is associated with diminished satiety. J. Clin. Endocrinol. Metab..

[66] Liu G., Zhu H., Lagou V., Gutin B., Stallmann-Jorgensen I.S., Treiber F.A., Dong Y., Snieder H. (2010). FTO variant rs9939609 is associated with body mass index and waist circumference, but not with energy intake or physical activity in European- and African-American youth. BMC Med. Genet..

[67] Sonestedt E., Roos C., Gullberg B., Ericson U., Wirfält E., Orho-Melander M. (2009). Fat and carbohydrate intake modify the association between genetic variation in the *FTO* genotype and obesity. Am. J. Clin. Nutr..

[68] Hainer V., Kunesova M., Bellisle F., Parizkova J., Braunerova R., Wagenknecht M., Lajka J., Hill M., Stunkard A. (2006). The Eating Inventory, body adiposity and prevalence of diseases in a quota sample of Czech adults. Int. J. Obes..

[69] Rideout C.A., Barr S.I. (2009). “Restrained eating” *vs.* “trying to lose weight”: How are they associated with body weight and tendency to overeat among postmenopausal women?. J. Am. Diet. Assoc..

[70] Savage J.S., Hoffman L., Birch L.L. (2009). Dieting, restraint, and disinhibition predict women’s weight change over 6 y. Am. J. Clin. Nutr..

[71] Foster G.D., Wadden T.A., Swain R.M., Stunkard A.J., Platte P., Vogt R.A. (1998). The eating inventory in obese women: Clinical correlates and relationship to weight loss. Int. J. Obes..

[72] Vogels N., Diepvens K., Westerterp-Plantenga M. (2005). Predictors of long-term weight maintenance. Obes. Res..

[73] Hill J.O., Wyatt H.R., Reed G.W., Peters J.C. (2003). Obesity and the environment: Where do we go from here?. Science.

[74] Bryant E.J., King N.A., Blundell J.E. (2008). Disinhibition: Its effects on appetite and weight regulation. Obes. Rev..

[75] Kilpeläinen T.O., Qi L., Brage S., Sharp S.J., Sonestedt E., Demerath E., Ahmad T., Mora S., Kaakinen M., Sandholt C.H. (2011). Physical activity attenuates the influence of *FTO* variants on obesity risk: A meta-analysis of 218,166 adults and 19,268 children. PLoS Med..

[76] Ahlberg A.C., Ljung T., Rosmond R., McEwen B., Holm G., Akesson H.O., Björntorp P. (2002). Depression and anxiety symptoms in relation to anthropometry and metabolism in men. Psychiatry Res..

[77] Davis C. (2009). Psychobiological traits in the risk profile for overeating and weight gain. Int. J. Obes..

[78] Rivera M., Cohen-Woods S., Kapur K., Breen G., Ng M.Y., Butler A.W., Craddock N., Gill M., Korszun A., Maier W. (2012). Depressive disorder moderates the effect of the *FTO* gene on body mass index. Mol. Psychiatry.

[79] Samaan Z., Anand S., Zhang X., Desai D., Rivera M., Pare G., Thabane L., Xie C., Gerstein H., Engert J.C. (2012). The protective effect of the obesity-associated rs9939609A variant in fat mass and obesity associated gene on depression. Mol. Psychiatry.

[80] Peeters A., Beckers S., Verrijken A., Roevens P., Peeters P., van Gaal L., van Hul W. (2008). Variants in the *FTO* gene are associated with common obesity in the Belgian population. Mol. Genet. Metab..

[81] Price R.A., Li W.D., Zhao H. (2008). *FTO* gene SNPs associated with extreme obesity in cases, controls and extremely discordant sister pairs. BMC Med. Genet..

